# Ligneous periodontitis exacerbated by Behçet’s disease in a patient with plasminogen deficiency and a stop-gained variant *PLG* c.1468C > T: a case report

**DOI:** 10.1186/s12903-023-03586-8

**Published:** 2023-11-08

**Authors:** Yuki Shinoda-Ito, Anna Hirai, Kazuhiro Omori, Hidetaka Ideguchi, Hideki Yamamoto, Fumino Kato, Kyoichi Obata, Tatsuo Ogawa, Keisuke Nakano, Takato Nakadoi, Eri Katsuyama, Soichiro Ibaragi, Tadashi Yamamoto, Hitoshi Nagatsuka, Akira Hirasawa, Shogo Takashiba

**Affiliations:** 1 Department of Pathophysiology-Periodontal Science, Faculty of Medicine, Dentistry and Pharmaceutical Sciences, Okayama University, 2-5-1 Shikata-Cho, Kita-Ku, Okayama, 700-8525 Japan; 2 Department of Periodontics and Endodontics, Division of Dentistry, Okayama University Hospital, 2-5-1 Shikata-Cho, Kita-Ku, Okayama, 700-8525 Japan; 3 Department of Clinical Genomic Medicine, Faculty of Medicine, Dentistry and Pharmaceutical Sciences, Okayama University, 2-5-1 Shikata-Cho, Kita-Ku, Okayama, 700-8558 Japan; 4 Department of Clinical Genomic Medicine, Okayama University Hospital, 2-5-1 Shikata-Cho, Kita-Ku, Okayama, 700-8558 Japan; 5 Department of Oral and Maxillofacial Surgery, Faculty of Medicine, Dentistry and Pharmaceutical Sciences, Okayama University, 2-5-1 Shikata-Cho, Kita-Ku, Okayama, 700-8525 Japan; 6 Department of Oral Pathology and Medicine, Faculty of Medicine, Dentistry and Pharmaceutical Sciences, Okayama University, 2-5-1 Shikata-Cho, Kita-Ku, Okayama, 700-8525 Japan; 7 Department of Nephrology, Rheumatology, Endocrinology and Metabolism, Faculty of Medicine, Dentistry and Pharmaceutical Sciences, Okayama University, 2-5-1 Shikata-Cho, Kita-Ku, Okayama, 700-8558 Japan; 8 The Center for Graduate Medical Education (Dental Division), Okayama University Hospital, 2-5-1 Shikata-Cho, Kita-Ku, Okayama, 700-8525 Japan

**Keywords:** Ligneous periodontitis, Plasminogen deficiency, *PLG*, Behçet's disease, Gingival hyperplasia

## Abstract

**Background:**

Plasminogen serves as the precursor to plasmin, an essential element in the fibrinolytic process, and is synthesized primarily in the liver. Plasminogen activation occurs through the action of plasminogen activator, converting it into plasmin. This conversion greatly enhances the fibrinolytic system within tissues and blood vessels, facilitating the dissolution of fibrin clots. Consequently, congenital deficiency of plasminogen results in impaired fibrin degradation. Patients with plasminogen deficiency typically exhibit fibrin deposits in various mucosal sites throughout the body, including the oral cavity, eyes, vagina, and digestive organs. Behcet's disease is a chronic recurrent systemic inflammatory disease with four main symptoms: aphthous ulcers of the oral mucosa, vulvar ulcers, skin symptoms, and eye symptoms, and has been reported worldwide. This disease is highly prevalent around the Silk Road from the Mediterranean to East Asia.

We report a case of periodontitis in a patient with these two rare diseases that worsened quickly, leading to alveolar bone destruction. Genetic testing revealed a novel variant characterized by a stop-gain mutation, which may be a previously unidentified etiologic gene associated with decreased plasminogen activity.

**Case presentation:**

This case report depicts a patient diagnosed with ligneous gingivitis during childhood, originating from plasminogen deficiency and progressing to periodontitis. Genetic testing revealed a suspected association with the *PLG* c.1468C > T (p.Arg490*) stop-gain mutation. The patient's periodontal condition remained stable with brief intervals of supportive periodontal therapy. However, the emergence of Behçet's disease induced acute systemic inflammation, necessitating hospitalization and treatment with steroids. During hospitalization, the dental approach focused on maintaining oral hygiene and alleviating contact-related pain. The patient's overall health improved with inpatient care and the periodontal tissues deteriorated.

**Conclusions:**

Collaborative efforts between medical and dental professionals are paramount in comprehensively evaluating and treating patients with intricate complications from rare diseases. Furthermore, the *PLG* c.1468C > T (p.Arg490*) stop-gain mutation could contribute to the association between plasminogen deficiency and related conditions.

## Background

Plasmin is a proteolytic enzyme crucial to the fibrinolytic system and is responsible for dissolving thrombi. In its inactive form, plasminogen circulates in the blood and is synthesized and secreted by the liver. Plasminogen is converted to plasmin by mediating plasminogen activators, enhancing the fibrinolytic process [1, 2]. Plasminogen deficiency, a rare genetic disorder, results in the absence of plasminogen, leading to reduced plasmin levels and consequent fibrin accumulation. Around 12,000 cases of plasminogen deficiency have been estimated globally, with conjunctivitis (81%) being the most common manifestation. Notably, 30% of patients present with ligneous periodontitis, associated with periodontal tissue destruction and tooth loss. Other affected areas encompass the respiratory tract and ears, with symptoms often more severe in females than males [3]. Surgical removal of gingival lesions has been reported to induce rapid regeneration, although progressive bone resorption frequently results in tooth loss [4–9]. Its pathology has been specified as one of the systemic diseases and conditions that affect the periodontal attachment apparatus in the consensus report established by the European Federation of Periodontology and the American Academy of Periodontology [10, 11].

Plasminogen deficiency is categorized into two types: true plasminogen deficiency (type I) and dysplasminemia (type II). Type I exhibits reduced plasminogen antigen and activity, while type II presents with normal or slightly decreased plasminogen antigen levels and significantly reduced activity [6]. Genetic mutations in the plasminogen gene (*PLG*), such as the Ala620Thr missense mutation and Asp219Asn mutation, have been linked to type I plasminogen deficiency and implicated in its pathogenesis [12, 13].

Comprehensive documentation of ligneous periodontitis has been well summarized recently [14]. This disease exclusively occurs in patients of type I plasminogen deficiency at the median age of 12.5 years at diagnosis. The marginal gingival tissues where teeth exist in both maxilla and mandibular are affected commonly more in females than males. Surgical excision of gingival hyperplasia lesion does not make long-term favorable prognosis due to the reason of reoccurring those excised gingiva rapidly. Moreover, application such as corticosteroids and heparin had been attempted to patients with poor effects on swelled gingiva. Mucosal control by replenishment of recombinant plasminogen would be definite, however this treatment has not been accepted majorly and the prognosis is still unclear. Thus, no infallible treatment and remedy for this disease have not been established at this moment. Consequently, to maintain oral hygiene carefully is the standard treatment for ligneous periodontitis.

Behçet’s disease, a recurrent systemic inflammatory disorder with an unknown cause, prevailing along the historic Silk Road connecting the Mediterranean and East Asia. Turkey has the highest prevalence at 80–370 per 100,000 population, while rates in Japan, Korea, China, Iran, and Saudi Arabia range from 13.5 to 20 per 100,000 [15]. The disease typically initiates in the thirties, marked by recurring symptoms followed by remission periods. Manifestations encompass the skin, mucous membranes, blood vessels, and nervous system. Mucocutaneous and ocular symptoms are prominent, pivotal features [16]. Medications like colchicine, prednisolone (PSL), and infliximab are commonly administered [17]. Genetic and environmental factors contribute to Behçet’s disease, with a noted association between the human leukocyte antigen HLA-B51 and the disease. This genetic marker serves as a diagnostic criterion [16, 18]. Observations of oral conditions and bacteria, specifically *Streptococcus sanguinis*, suggest a possible link between oral health and Behçet’s disease [19, 20].

This case report presents the case of a patient diagnosed with plasminogen deficiency in childhood, that progressed to ligneous gingivitis and subsequent periodontitis with significant tissue destruction in adolescence. Genetic testing uncovered a novel *PLG* gene variant characterized by a stop-gain mutation. This unique variant might have implications for the etiology of ligneous periodontitis. Additionally, the patient developed systemic inflammation due to Behçet’s disease, accelerating periodontal tissue destruction. To our knowledge, this is the first reported coexistence of plasminogen deficiency and Behçet’s disease. This multifaceted case offers valuable insights for future periodontal treatment in exceptionally rare cases involving both conditions.

## Case report

### Diagnosis of plasminogen deficiency and ligneous periodontitis

In 2019, a 20-year-old woman was referred to the Division of Dentistry, Department of Periodontics and Endodontics, Okayama University Hospital in Japan by a general practice dentist. The referral aimed to seek specialized periodontal treatment due to significant periodontal tissue destruction caused by ligneous periodontitis associated with plasminogen deficiency. Herein, we present the sequence of events leading to the diagnosis of plasminogen deficiency during the patient’s childhood and her subsequent presentation to our department in 2019.

**Fig. 1:**
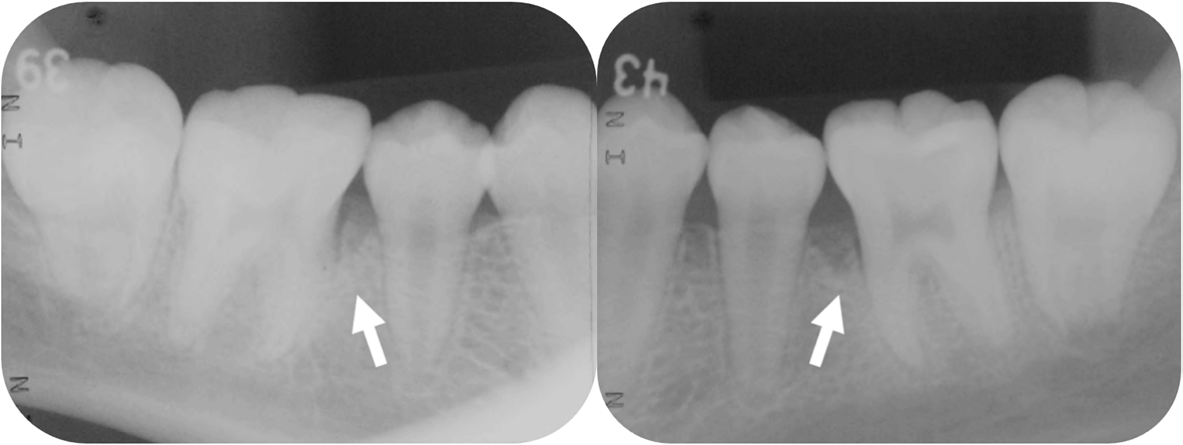
Dental radiographs taken at 12 years of age in 2012. Vertical bone resorption was observed on the mesial side of the bilateral mandibular first molars (teeth number 36 and 46)

The patient initially received a diagnosis of ligneous gingivitis linked to plasminogen deficiency at the Department of Oral and Maxillofacial Surgery, Okayama University Hospital, in 2008, when she was just nine years old. At that time, her blood plasminogen activity was as low as 10%, and pseudomembranous white lesions were evident on the gingiva and eye [21]. Dental radiographs taken in 2012, when she was 12 years old, unveiled signs of vertical bone resorption in the lower first molar, indicative of ligneous periodontitis accompanied by alveolar bone destruction (Fig. 1). Despite receiving regular supportive periodontal therapy (SPT) at her referring dental office for eight years, she was referred back to Okayama University Hospital in 2019 due to the remarkable extent of alveolar bone destruction for her age.

**Fig. 2:**
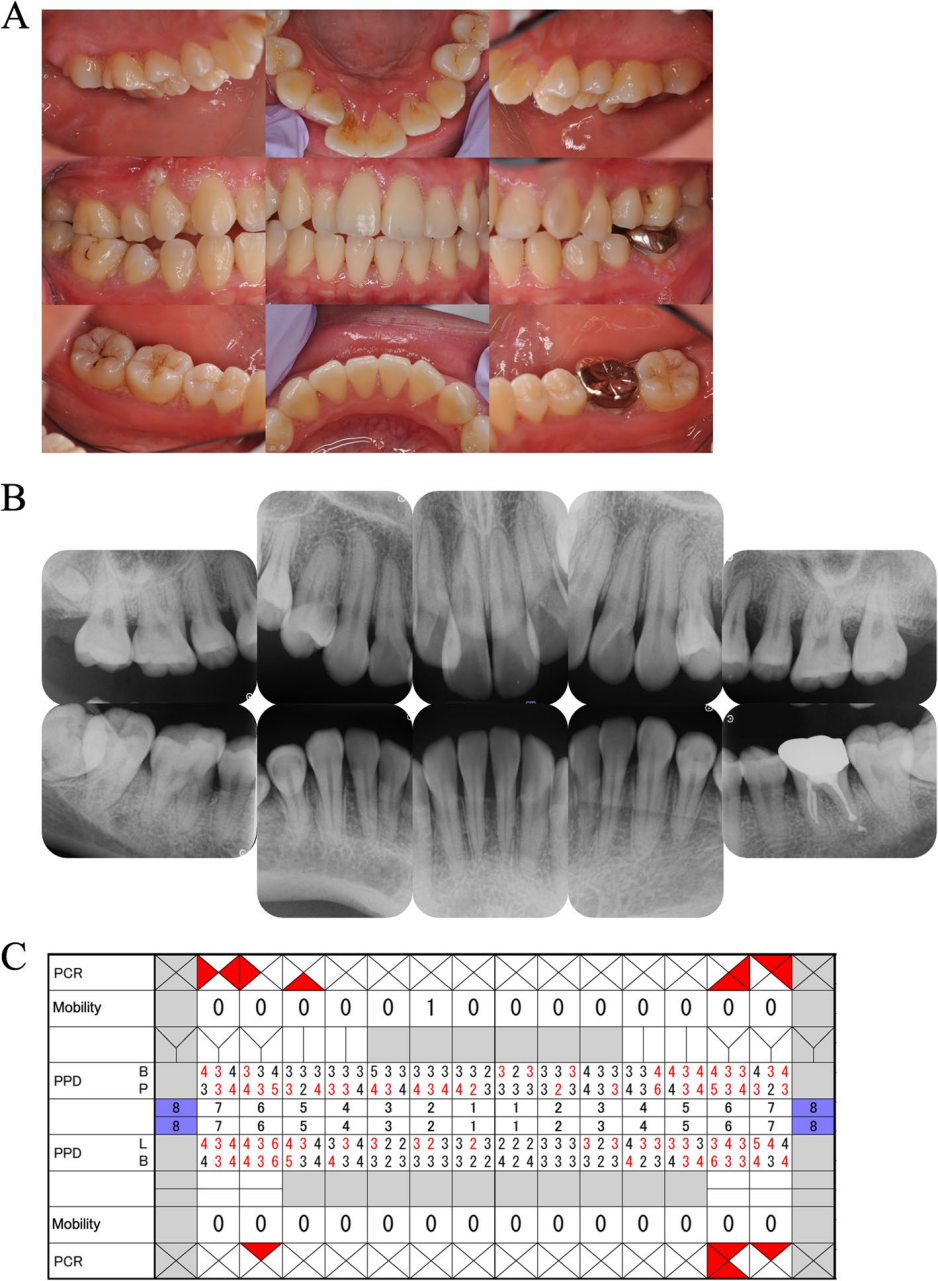
Intraoral photographs (**A**) and dental radiographs (**B**) from a return visit in 2019. **A** The marginal gingiva was swollen and erythematous, and the interdental papillary gingiva was hyperplastic and swollen. The hyperplasia of the buccal marginal gingiva was particularly pronounced at 11, 14, and 36. In the central occlusal position, only the molars were in occlusal contact. **B** Radiographically, horizontal bone resorption of 1/4 to 1/3 of the root and vertical bone resorption of the molars were observed. 18, 38 and 48 were impacted. **C** Although oral hygiene was relatively good, periodontal pockets over 4 mm and BOP-positive areas were existed mainly in the molar regions

**Fig. 3:**
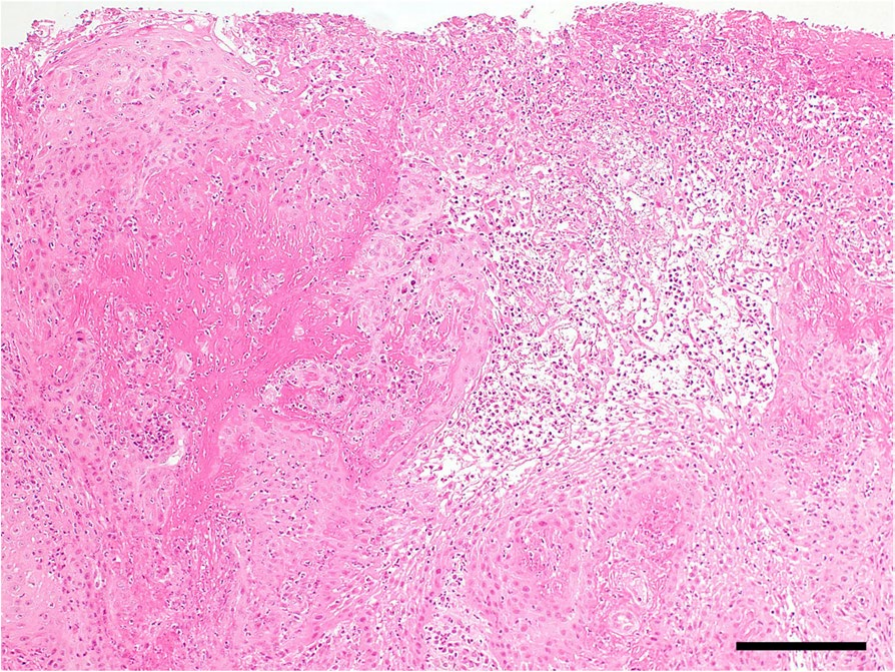
Histopathological findings of the gingival specimens. Fibrin precipitation and neutrophilic infiltration within the thickened stratified squamous epithelium were observed in the tissue sections of hyperplastic gingiva hematoxylin–eosin (**H**-**E**) staining. Scale bar: 200 µm

Upon re-referral in 2019, her plasminogen activity was less than 25%, confirming the diagnosis of plasminogen deficiency. However, other markers, such as prothrombin time-international normalized ratio (PT-INR) and D-dimer, were within normal ranges, indicating no predisposition to bleeding. In terms of oral health, despite maintaining good oral hygiene (O’Leary plaque control record (PCR): 11.6%), the patient exhibited marginal gingival redness in the molar region, gingival hyperplasia on the buccal gingiva of the maxillary anterior teeth and mandibular molars, and deep periodontal pockets primarily in the molar area (4 mm < probing pocket depth (PPD): 33.4%; periodontal inflamed surface area (PISA): 1,025 mm^2^). Dental radiographic examination revealed horizontal bone resorption of approximately one-third of the root and vertical bone resorption in all molars (Fig. 2). Histological examination of the gingival hyperplastic area demonstrated fibrin deposition and epithelial degeneration with neutrophilic infiltration (Fig. 3). Based on the above intraoral and radiographic findings, blood test results, and histological tissue examination, she was again diagnosed with ligneous periodontitis (Stage III, Grade C).

### Germline testing related to plasminogen deficiency

**Fig. 4:**
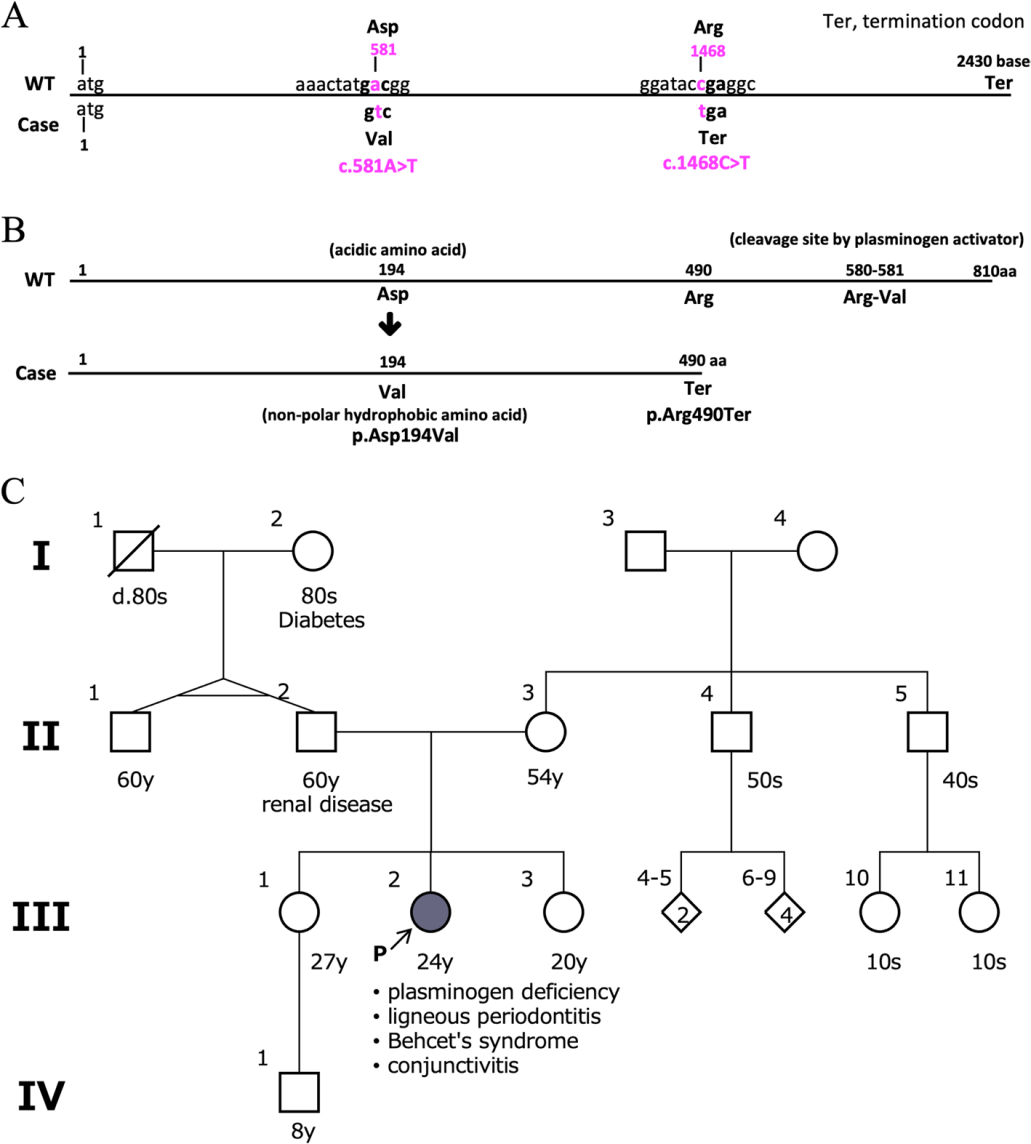
Results of genetic testing. **A** DNA sequences of *PLG* wild type (WT) and two variants of *PLG* detected in this case. The upper part of the horizontal line shows the nucleotide sequence of WT *PLG*, and the lower part shows the sequence of *PLG* in this patient. The nucleotide substitution *PLG* c.581A > T represents a missense mutation, and the nucleotide substitution *PLG* c.1468C > T represents a stop gain mutation, introducing a termination codon (tga). **B** Amino acid sequences of wild-type (WT) *PLG* and *PLG* variants were putatively produced in this case. The upper part shows the amino acid sequence of WT *PLG*, and the lower part shows the sequence in this patient. The number of amino acids (aa) is indicated at the top of each horizontal line. The total number of amino acids in WT *PLG* is 810, while in this patient, it is 490, indicating a shorter sequence compared to WT. The plasminogen activator cleavage site, located at positions 580–581 in WT *PLG*, is absent in this patient. Ter: termination codon. aa: amino acid. **C** Pedigree of the patient’s family. The proband is indicated by an arrow. The black circle represents an individual with plasminogen deficiency, ligneous periodontitis, conjunctivitis, and Behcet's disease. Other symptoms are indicated by unfilled circles or squares. The shaded line indicates the deceased. d: Dead

We analyzed genomic sequences using targeted next-generation sequencing (NGS) with the hybrid capture method to explore potential germline variants underlying plasminogen deficiency. The analysis focused on a several-gene panel, including *F12* and *PLG*, associated with plasminogen deficiency. Rare variants in *F12* and *PLG* were identified through comparison with population database such as the Genome Aggregation Database (gnomAD). Specifically, the *F12* variant A343P had been registered with conflicting interpretations of pathogenicity in the ClinVar database, with an allele frequency of 3.00 × 10^–3^. *PLG* c.581A > T (p.Asp194Val) was registered as benign in the ClinVar database (Variation ID: 780,122), with an allele frequency of 7.68 × 10–4. *PLG* c.1468C > T (p.Arg490*) was not reported in the ClinVar database, and its allele frequency in the gnomAD database was 1.19 × 10^–5^ (Table 1). The *PLG* gene exhibited a c.581A > T missense mutation in this patient and a c.1468C > T stop-gain mutation (Fig. 4A). These mutations resulted in the truncation of the *PLG* protein and the loss of its plasminogen activator cleavage site (Fig. 4B). The patient had no family history of plasminogen deficiency or Behçet’s disease (Fig. 4C).

**Table 1:** F12 and PLG variants

Variant	Database	Population	Allele number	Allele frequency
*F12* A343P	GEM-J WGA	Japanese	14,792	5.48 × 10^–2^
	ToMMo	Japanese	77,440	5.77 × 10^–2^
	gnomAD	East Asian	18,864	3.40 × 10^–2^
	gnomAD	Global	254,118	3.00 × 10^–3^
*PLG* D194V	GEM-J WGA	Japanese	15,196	2.63 × 10^–3^
	ToMMo	Japanese	77,438	2.67 × 10^–3^
	gnomAD	East Asian	19,946	1.06 × 10^–2^
	gnomAD	Global	282,726	7.68 × 10^–4^
*PLG* R490*	GEM-J WGA	Japanese	-	-
	ToMMo	Japanese	-	-
	gnomAD	East Asian	18,394	0
	gnomAD	Global	251,406	1.19 × 10^–5^

Allele frequencies of *F12* and *PLG* variants in the general population are shown. Allele numbers are the total number of alleles analyzed in each database. All data were obtained by accessing each database in June 2023. ^*^: stop codon

### Treatment for ligneous periodontitis

**Fig. 5:**
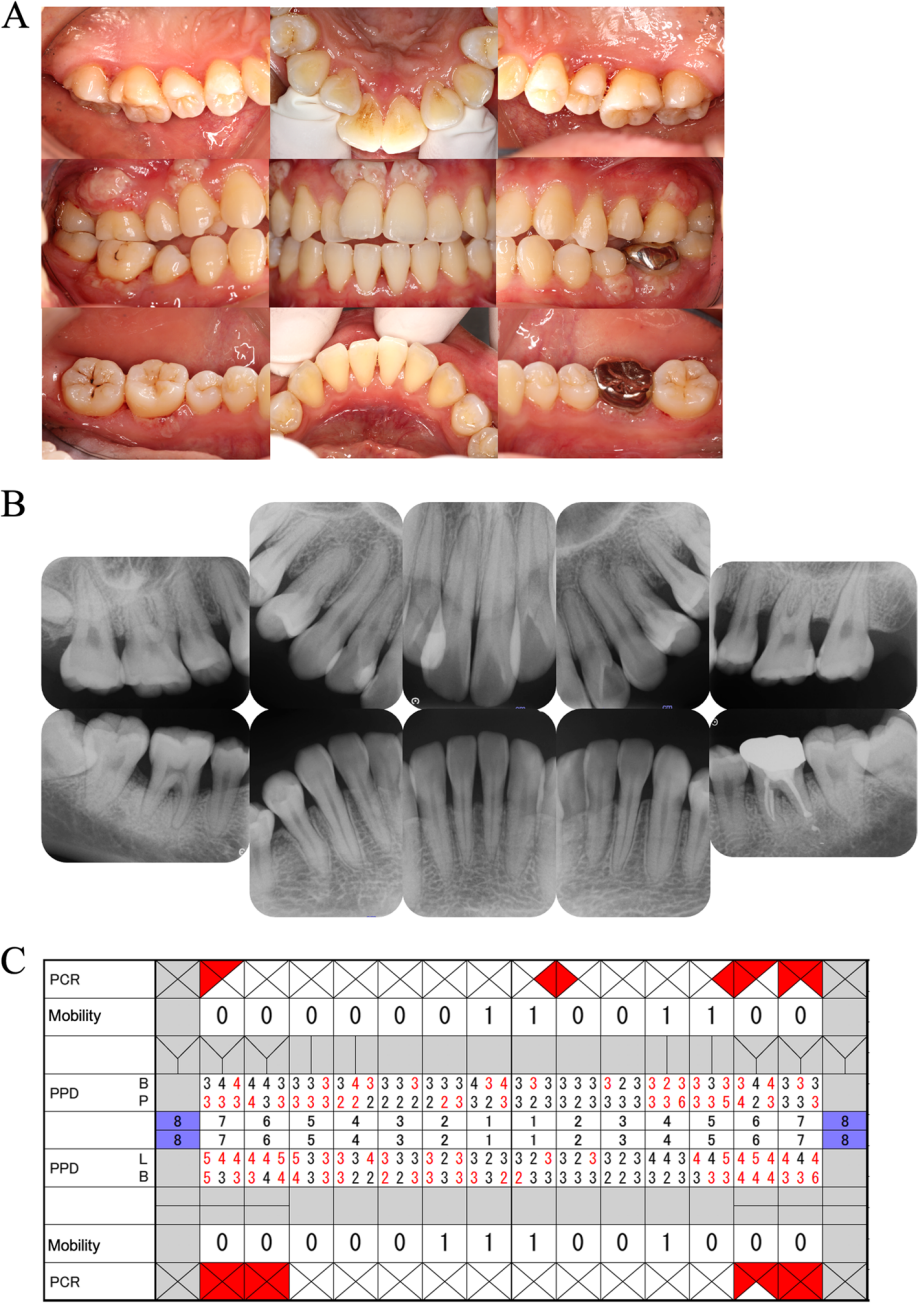
Intraoral photographs and dental radiographs at the time of transition to SPT phase in 2021. **A** Gingival hyperplasia showed a proliferative trend compared to the patient's return visit in 2019, accompanied by persistent marginal gingival erythema and swelling. Hyperplasia of the buccal marginal gingiva was particularly marked at the maxillary central incisors, premolars, and molars of both jaws. **B** There were no significant changes in bone resorption compared to the patient's return visit in 2019. **C** Although the patient maintained relatively good oral hygiene, high BOP-positive rate was consistent with deep periodontal pockets mainly in the molar region

Initial periodontal treatment for the patient involved non-surgical scaling and root planning (SRP) to eliminate the subgingival source of infection. Additionally, the patient used a mouthpiece to alleviate occlusal forces. Subsequent SPT was provided at short intervals from 2021 to stabilize the periodontal condition, although gingival hyperplasia and swelling showed minimal improvement. Periodontal surgery was not considered due to the potential for increased fibrin deposition with gingival incision. The patient’s oral condition at SPT was as follows: PCR 22.3%, BOP 49.4%, 4 mm < PPD 23.8%, PISA 981.4 mm^2^ (Fig. 5).

### Onset of Behçet’s disease

**Fig. 6:**
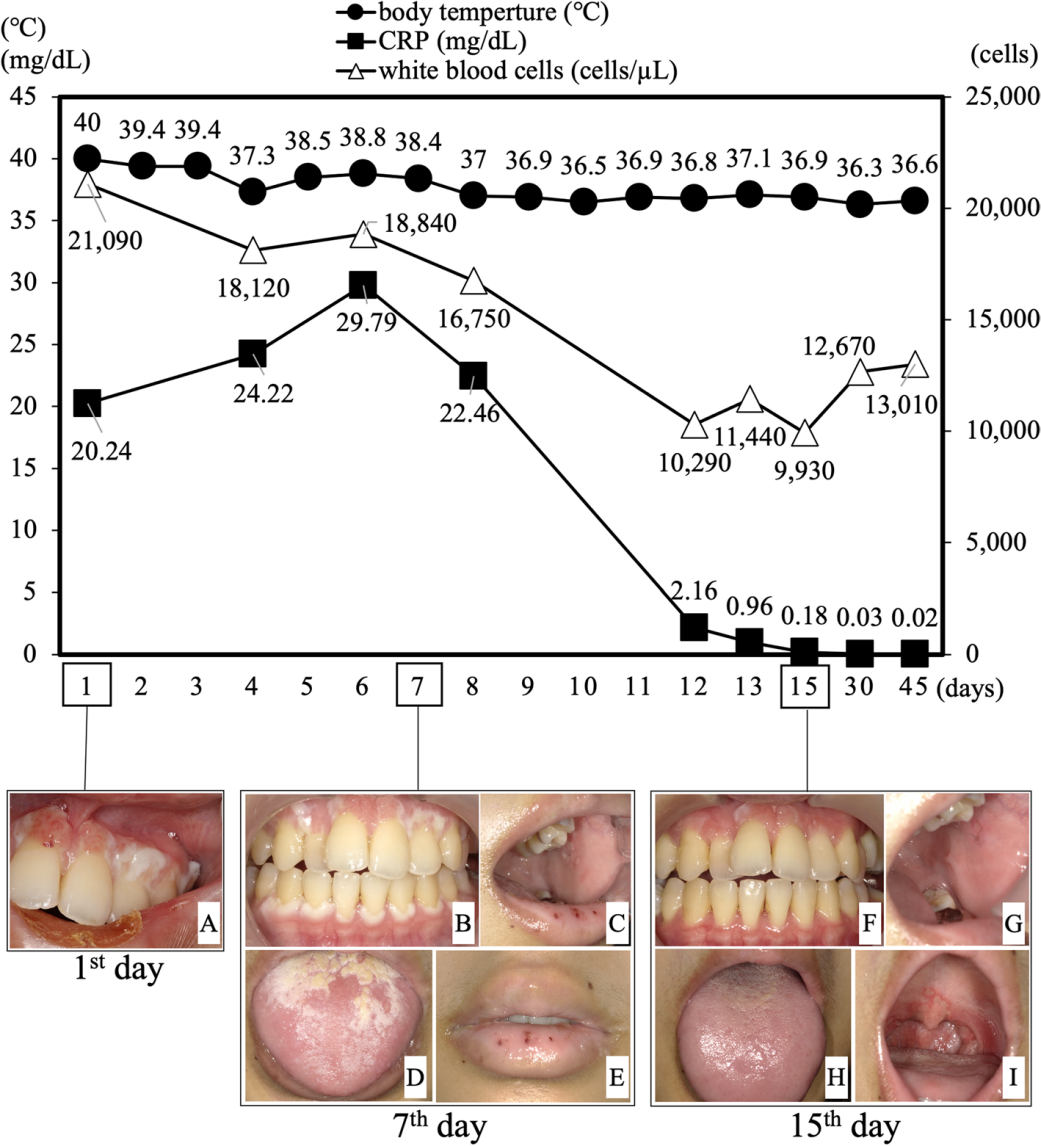
Changes in blood test results and intraoral photographs during hospitalization. The graphs depict the temporal changes in temperature (●), C-reactive protein level (∆), and white blood cell (WBC) count (■) throughout the hospitalization period. The horizontal axis of the graph shows the number of days since admission, while the left vertical axis shows the temperature (Celsius) or CRP level (mg/dL), and the right vertical axis shows the WBC count. The lower section of the graph corresponds to the first day of hospitalization (left; A), seven days after hospitalization (middle; B-E), and fifteen days after hospitalization (right; **F**-**I**). **A** Gingival hyperplasia was thickened and progressed on maxillary incisors on the first day of hospitalization. **B** Gingival hyperplasia on the seventh day of hospitalization remained the same as on the first day. **C** Multiple aphthous ulcers were seen on the buccal mucosa. **D** The tongue was covered with a thick biofilm. **E** Multiple aphthous ulcers were also seen on the lips. **F** Fifteen days after admission, the gingival hyperplasia had improved to the same level as at the time of SPT transition. **G** Multiple aphthous ulcers on the buccal mucosa disappeared. **H** Biofilm on the tongue almost disappeared. **I** Pharyngeal erythema remained

Despite maintaining stability systemically and orally, the patient presented to the Department of General Medicine, Okayama University Hospital, in 2022 with complaints of fever (body temperature (BT) 40.0 °C), diarrhea, and general malaise. Blood tests revealed a significantly elevated white blood cell (WBC) count (21.09 × 10^3^ cells/µL) and C-reactive protein (CRP) level (20.24 mg/dL), leading to urgent hospital admission for further evaluation and treatment. Initial suspicion was directed towards infectious diseases, prompting the initiation of antibiotic therapy (tazobactam/piperacillin hydrate, 4.5 g × 3 times/day). However, the patient rapidly developed additional systemic symptoms, including hematochezia, vulvar ulcers, and an erythema nodosum-like rash on the extremities. In the oral cavity, marginal gingival hyperplasia, erosions, and multiple aphthous stomatitis were observed. Additionally, the tongue was coated with a thick biofilm (Fig. 6). Subsequently, she was diagnosed with intestinal Behçet’s disease based on the primary symptoms of erythema nodosum-like skin rash on the extremities, recurrent aphthous ulcers on the oral mucosa, and atypical genital ulcer. Secondary symptoms, including vasculitis and gastrointestinal involvement, were confirmed through histological examination of the erythematous nodular area. Seven days after hospitalization, the presence of HLA-B51 antigens was detected, further supporting this diagnosis.

### Treatment for Behçet’s disease

**Fig. 7:**
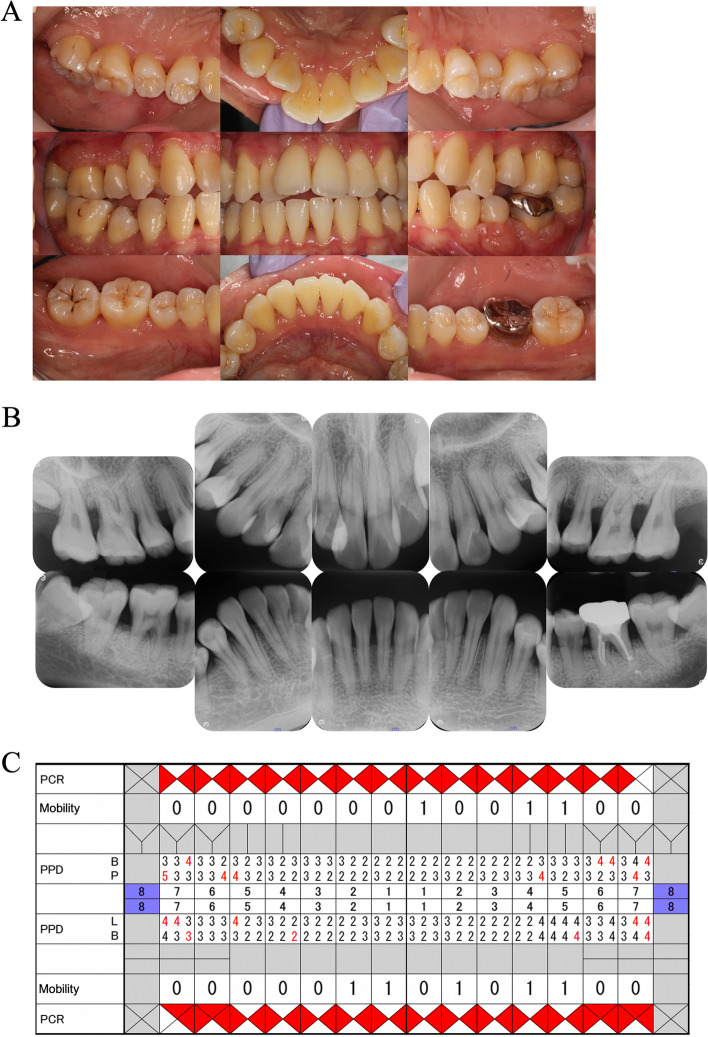
Intraoral photographs (**A**) and dental radiographs (**B**) on the 45^th^ day after hospitalization (discharge date). **A** Gingival hyperplasia exhibited a notable reduction and improvement from the initial day of hospitalization, reaching a state similar to that observed upon transfer to SPT. However, persistent hyperplasia was still evident in the same regions where pronounced hyperplasia was observed during the transition to SPT in 2021. **B** Horizontal bone resorption in the molars showed further progression compared to the previous visit, as evidenced by radiographic findings displaying bone-like structures between 45 and 46. **C** Oral hygiene status deteriorated as systemic condition worsened, however deep periodontal pockets and BOP-positive rates declined

Prompt administration of oral prednisolone (PSL) at 1 mg/kg/day was initiated upon the diagnosis of Behçet’s disease. Oral hygiene was maintained during hospitalization, and mucosal treatment was provided using glycerin mouthwashes. On the 15^th^ day after hospitalization, with reduced systemic inflammation due to PSL treatment (CRP 0.18 mg/dL, WBC 9,330 cells/µL, BT 36.9 °C), aphthous ulceration, gingival hyperplasia, and erosion areas showed improvement, and the biofilm on the tongue disappeared. However, redness in the pharyngeal region persisted. Consequently, the PSL dosage was gradually reduced to 30 mg/day. On the 45^th^ day of hospitalization, the patient’s overall condition had significantly improved, leading to her discharge from the hospital (CRP 0.02 mg/dL, WBC 13,101 cells/µL, BT 36.6 °C). Around the same time, aphthous ulceration in the oral cavity had resolved. Gingival hyperplasia had decreased to a level similar to when she transferred to SPT, though gingival recession seemed to have progressed (PCR 51.8%, BOP 10.7%, 4 mm < PPD 16.7%, PISA 226.4 mm^2^). Conversely, dental radiographs revealed progressive horizontal alveolar bone resorption (bone resorption of approximately two-thirds of the root of the tooth) compared to the previous assessment (Fig. 7).

Following her discharge, the patient initiated the treatment with the anti-tumor necrosis factor (TNF)-α antibody drug adalimumab at 40 mg every two weeks. This treatment aimed to decrease reliance on steroids and provide symptom relief through the anti-inflammatory effects of the medication. The patient experienced intermittent flares and remissions of systemic inflammation after hospital discharge, although to a lesser extent than during the initial hospitalization period.

## Discussion

Ligneous periodontitis in this patient, caused by plasminogen deficiency, was rapidly exacerbated by acute systemic inflammation due to the onset of Behçet’s disease. Concurrently, the presence of aphthous stomatitis, a symptom of Behçet’s disease, significantly impeded the patient’s ability to eat and swallow.

Plasminogen, a precursor to plasmin, plays a crucial role in the fibrinolytic system by dissolving fibrin. Consequently, local fibrin deposition occurs in patients with plasminogen deficiency. As fibrin functions as a proinflammatory cytokine, it has been reported that fibrin deposition on the oral mucosa leads to periodontal tissue destruction and early progression of periodontitis [9, 22]. Consistent with these reports, we observed the development of ligneous periodontitis, characterized by significant periodontal tissue destruction, in this patient from a young age. Indigenous bacteria have been found to induce extravascular fibrin deposition in the oral mucosa, where deposited fibrin binds to neutrophils and enhances their effector function, ultimately leading to tissue destruction [23, 24]. The lack of plasminogen may result in excessive fibrin activity in patients with plasminogen deficiency, further contributing to periodontal tissue destruction [7, 25].

Previous studies have reported various genetic mutations associated with plasminogen deficiency, including G142R, G176D, T181P, D219N, R234H, P285T, R306H, N307I, T319_N320insN, T352I, K378X, S441R, E455fsX493, IVS11-2 A/G, P491R, A505V, Q540X, L650fsX652, P744S, C765G, and R776H [6] and A620T [11]. As for the genetic variants identified in this patient, *F12* A343P is considered to have low pathological significance based on its allele frequency in the Japanese database (ToMMo). The *PLG* variants found in this patient are rare, with *PLG* D194V being classified as benign in the ClinVar database and *PLG* R490* not previously reported in the Japanese population. In this patient, the *PLG* R490* mutation results in the loss of the plasminogen activator cleavage site located at amino acid sequence 580–581, which is critical for normal plasminogen function. Thus, it is predicted that these missense and stop-gain mutations are responsible for the plasminogen deficiency observed in this patient. Plasminogen deficiency is an autosomal latent genetic disease; however, no other family members of this patient have been affected. The patient's siblings and mother continue to undergo SPT at private dental offices, and no severe progression of periodontitis has been observed. The patient's father, who has experienced multiple tooth loss, utilizes dentures for both the maxilla and mandible. Nonetheless, no genetic testing has been conducted within the family, warranting further investigation of the genetic background for future studies.

Behçet's disease is an intractable inflammatory disorder characterized by recurrent acute inflammation in multiple organs. Oral aphthous ulcers, a prominent symptom of Behçet's disease, occur in up to 90% of patients and are often the initial manifestation of the disease. These ulcers primarily affect the oral mucosa, including the lips, buccal mucosa, gingiva, and palate, often causing pain during eating and swallowing [26]. The patient in this study also experienced periods of difficulty with contact and swallowing due to contact pain caused by recurrent aphthous ulcers. Behçet's disease is multifactorial and involves infectious and environmental factors, such as herpes viruses and microorganisms like *Streptococcus sanguis* [20], in addition to a genetic predisposition, particularly the HLA-B51 allele [16, 18, 26]. Host-derived heat shock proteins (HSPs), which cross-react with bacterial HSPs, are hypothesized to act as autoantigens and trigger an autoimmune response leading to inflammation. HSPs also play a role in inflammatory cytokines, including TNF-α and interleukin (IL) [18, 27, 28]. Anti-TNF monoclonal antibodies have shown particular effectiveness in treating intestinal Behçet's disease [29]. In this patient, an anti-TNF-α monoclonal antibody was administered. While TNF-α monoclonal antibody therapies have demonstrated efficacy in treating Behçet's disease, patients receiving these treatments are at risk of opportunistic infections due to immunosuppression [30, 31]. Early control of oral infections in patients with Behçet's disease has been reported to contribute to improved disease management and the effectiveness of anti-TNF-α therapy [32]. Hence, meticulous oral hygiene management is crucial for patients with Behçet's disease.

Regarding to relationships between other autoimmune diseases and periodontitis, there is a report that patients with periodontitis has higher risk of rheumatoid arthritis, Sjogren's syndrome and psoriasis [33]. Other reports suggested that periodontal treatment improved the marker of rheumatoid arthritis [34, 35]. These reports indicate that some autoimmune diseases and periodontitis may have interactions with each other. Supportively, some researchers suggested that single nucleotide polymorphisms (SNPs) of proinflammatory cytokines such as IL-1 and TNF-α genes related to periodontitis and autoimmune diseases, especially Behçet’s disease [36, 37]. Furthermore, IL-33 which is the new member of IL-1 may relate to autoimmune diseases and periodontitis because of its ability to activate the secretion of proinflammatory cytokines via nuclear factor kappa-B [38]. The whole genome sequences of this patient will be useful to evaluate SNPs of proinflammatory cytokines comprehensively in the future.

Although this patient exhibited periodontal tissue destruction from a young age due to ligneous periodontitis, initial periodontal treatment and regular SPT at short intervals maintained relatively stable periodontal conditions. However, with the onset of Behçet's disease, systemic inflammation and subsequent decline in overall health led to a rapid progression of periodontitis within a short time frame. Although there were no signs of stomatitis in the oral cavity from the patient's initial visit to our clinic until the onset of Behçet's disease, it is plausible that these symptoms were obscured by the gingival hyperplasia associated with ligneous periodontitis. Further approaches to ligneous periodontitis with administrating the recombinant plasminogen to this patient was considered before the onset of Behçet's disease. However, the patient still repeats exacerbation and remission of Behçet's disease symptoms so that is not in the condition that new approach to be attempted. *Porphyromonas gingivalis* (*P. gingivalis*) was found in high abundance within the deep periodontal pockets of this patient, with corresponding elevated serum antibody titers (Data not shown). This suggests a potential correlation between the patient's heightened susceptibility to *P. gingivalis* and the pathogenesis of her periodontitis. Additionally, oral administration of PSL for the treatment of Behçet's disease temporarily alleviated the exacerbated ligneous periodontitis (gingival proliferation and hyperplasia) as a secondary effect, indicating its potential efficacy as a treatment for ligneous periodontitis. However, long-term administration of PSL should be approached cautiously due to the associated risk of inducing osteoporosis.

## Conclusions

When multiple rare diseases manifest concurrently, as in this patient's case, identifying the primary disease can be time-consuming due to the complex nature of the immune system. Therefore, collaborative efforts between medical and dental professionals are crucial for conducting a comprehensive examination and treatment of such patients from various perspectives. Furthermore, discovering the *PLG* c.1468C > T (p.Arg490*) stop-gain mutation may enhance our understanding of the relationship between plasminogen deficiency and the development of associated diseases.

## Data Availability

Data will be made available by corresponding author on request.

## Abbreviations

BT: Body temperature
CRP: C-reactive protein
H-E: Hematoxylin–eosin
HSPs: Heat shock proteins
IL: Interleukin
NGS: Next generation sequencing
PCR: Plaque control record
*P. gingivalis*: *Porphyromonas gingivalis*
PISA: Periodontal inflamed surface area
*PLG*: Plasminogen gene
PPD: Probing pocket depth
PSL: Prednisolone
PT-INR: Prothrombin time-international normalized ratio
SPT: Supportive periodontal therapy
SRP: Scaling and root planning
SNPs: Single nucleotide polymorphism
TNF: Tumor necrosis factor
WBC: White blood cell
